# Improving the Quality of Residency Programs in Family and Community Medicine

**DOI:** 10.11606/s1518-8787.2023057004822

**Published:** 2023-09-14

**Authors:** Lucas Gaspar Ribeiro, Eliana Golbfarb Cyrino, Antônio Pazin-Filho

**Affiliations:** I Universidade de São Paulo Faculdade de Medicina de Ribeirão Preto Departamento de Clínica Médica Ribeirão Preto SP Brasil Universidade de São Paulo. Faculdade de Medicina de Ribeirão Preto. Departamento de Clínica Médica. Ribeirão Preto, SP, Brasil; II Universidade Estadual Paulista “Júlio de Mesquita Filho” Faculdade de Medicina de Botucatu Departamento de Saúde Pública Botucatu SP Brasil Universidade Estadual Paulista “Júlio de Mesquita Filho”. Faculdade de Medicina de Botucatu. Departamento de Saúde Pública. Botucatu, SP, Brasil

**Keywords:** Family and Community Medicine, Boarding and Residency, Evaluation of Research Programs and Instruments

## Abstract

**OBJECTIVE:**

To develop and present an instrument to evaluate and monitor the quality of medical residency programs in residencies in family and community medicine (FCM) based on preceptors and residents, considering the insertion of the health network program.

**METHOD:**

The instrument was developed in three stages: 1) interview with the preceptors of FCM; 2) literature review; and 3) production, adequacy, and approval of the evaluation instrument by renowned professionals of the Brazilian FCM. The third stage included 9 people and used the Delphi technique with 80% agreement. For the qualitative results, Bardin’s Content Analysis was used.

**RESULTS:**

In all, there were five evaluation cycles to adapt the proposed recommendations, with the elimination of one item and weighting, with a results analysis methodology of 10 resulting items, reaching an expected matrix for organizing residency programs in the health network, divided into 3 domains: Organization of the Unit, Human Resources, and Preceptor-resident relationship.

**CONCLUSION:**

An instrument for evaluating and monitoring residency programs in family and community medicine can be a tool to facilitate program managers and allow evaluation and monitoring, continuously qualifying them.

## INTRODUCTION

Medical residency is considered the best strategy for training new medical specialists, considered training after completing graduation. Some authors report that it began in the United States with medical and surgical clinics in the late 19th century. In Brazil, it is legally considered a postgraduate course, a specialization modality, focused on in-service training, existing since 1944^1^.

Residencies in Family and Community Medicine (FCM) began in 1976^1^, showing a small expansion of vacancies until 2013 compared to the following period (2013–2021)^2^. As of Law 12,871/13, known as the *Mais Médicos* Law, the number of vacancies occupied increased from 206 in 2011 to more than 2,282 (an increase of 11.7 times), data from October 2021. The state of São Paulo had 62 medical residency programs (MRP) in MFC registered, with 37 programs with residents (405 residents and 1,366 vacancies, 29% occupancy), corresponding to 17.8% of the MFC residents in Brazil, being the state with the highest number^3^ in 2021. The data for 2021 were obtained from the Law on Access to Information^4^.

As much as the Family Health Strategy (FHS) is based on the presence of a FCM specialist as a medical professional, the country has approximately 7,000 FCM professionals^3^for more than 43,000 Family Health teams^5^. This discrepancy intensifies when observing their distribution, as they are mostly present in the South and Southeast, corresponding to 71.4% of specialists in Brazil and 46% of family health units^3,5^.

Amapá, Bahia, Maranhão, and Piauí have less than 1.1 specialists per 100,000 inhabitants, whereas Acre, Distrito Federal, Rio Grande do Sul, and Santa Catarina have between 4.9 and 8.7 specialists per 100,000 inhabitants^3^. To calculate the ideal ratio, it is possible to infer that each family and community doctor is responsible for 4,000 people^6^, that is, the country would need a ratio of 25 family and community doctors per 100,000 inhabitants. Thus, considering that in 2020, approximately 1,500 new specialists were trained in residency, maintaining the current volume of vacancies occupied, the country would only reach the number of specialists needed after two decades, without considering losses over time.

Given the scarcity and concentration of specialists, training depends on preceptors from other areas of activity, who are legally qualified for such a role, according to the specific legislation of the MRP-FCM, unlike other areas, which require expertise in the area of training as a prerequisite for the function^7^.

This probably implies great variability in training conditions. In addition to this situation, there is also a lack of information and standards for minimum conditions of a physical structure and adequate inputs to guarantee training. With this, the programs need guidelines for opening and expanding vacancies in an orderly manner and capable of supplying training, considering the uniqueness of FCM, which operates predominantly in the FHS/Primary Health Care (PHC).

For this, it is necessary to understand the service itself and evaluate it. This act involves issuing a value judgment based on a predetermined standard or an ideal reference, looking for flaws and correcting them^8^. Donabedian^11^ reinforces that the evaluation process requires continuous monitoring of the health service, seeking to detect and correct departures from standards, being able to evaluate structures, processes and results obtained. In view of these factors, this study proposed a matrix for evaluating and monitoring residency programs in FCM in Brazil, with a focus on PHC.

## METHODS

This is an evaluative, descriptive, and exploratory methodology study, starting in 2018 to 2021. It consists of quantitative and qualitative research, using an electronic form to obtain responses from participants and a literature review for complementation and characterization of the instrument presented at the end.

This study developed a matrix instrument to define the training conditions offered by medical residency programs in FCM based on the PHC structure. The construction was divided into three stages of development, starting with understanding the preceptor’s reality and ending with the proposition of an evaluative instrument with the possibility of nationwide application.

In the first stage, 132 FCM preceptors from the state of São Paulo were invited from February to November 2018, applying a Google Forms, with open and closed questions. All professionals were invited via e-mail to understand the professional’s characteristics and the work they perform. The complete results have been reported in a specific article^12^, which listed the strengths, opportunities, weaknesses, and threats (SOWT) that 67 preceptors perceive in their work and in the MRP-FCM.

With the answers obtained, the second stage began with a review of national and international literature, resolutions and ordinances on medical residency in general and FCM, comparing the literature with the preceptors’ perception, superimposing SWOT with the Donabedian triad^12^. The framework constructed allowed obtaining responses that could be evaluated in PHC, generating a matrix of recommendations on FCM residency programs, completing the second stage of the matrix to be validated nationally.

The third stage peer-validated the document, ensuring quality homogeneity, with the possibility of use in all regions. For that purpose, the instrument was presented to a group of FCM experts, using the Delphi method^13^. The presidents of all active FCM state associations and the president of the Brazilian Society of Family and Community Medicine were invited to participate, comprising 24 people. These were chosen by the authors considering their regional diversity and national representativeness. The president of the São Paulo state association was excluded for being the project’s author.

The Delphi questionnaire had the same structure for all questions: Description of the evaluated item, questions about the pertinence and permanence of the item in the final questionnaire (yes and no), 0-10 Likert scales about the relevance of the item and what score or value this item should receive in the program evaluation (0 to 10). Open questions about wording suggestions and comments on the score and topic in question were also included in all items to improve the item. For the items to be maintained, a consensus among the judges had to be reached, the cut-off value for maintenance was 80%, with lower values for non-approval of the item.

For open questions, with text suggestion and verification sources, Bardin’s Analysis was used, allowing to adjust the writing, justify exclusions, and merge items if necessary.

Both surveys, with both the preceptor and the judges, were approved by the Research Ethics Committees, under protocols CAAE 78853317.0.0000.5411 and 30805420.5.0000.5440.

At the end of the discussion cycles, the recommendations were reassessed by the authors, organized, and compared with current legislation.

## RESULTS

Phase 1 responses showed that 70% of respondents were FCM specialists and more than 90% had some specific training in preceptorship. Although 62% (42 people) considered themselves quite satisfied with their performance, 27 people reported great difficulty at work.

The rewards most frequently reported by preceptors were: qualification in the area, keeping up-to-date, personal qualification, and participating in a transformation process. The challenges were: excessive demand versus difficulty in teaching time, undervaluation of the specialty, difficulty in the relationship with local management and difficulty in the teaching process, insufficient physical space for education, and difficulty in organizing the unit’s agenda.

By associating the preceptor’s point of view with the national and international literature on medical residency and on the specific area, a matrix of recommendations for FCM residency programs (stage 2) was produced. It was presented to the judges for continuing the construction process. Altogether, it took five evaluation cycles among the judges to obtain at least 80% agreement, with nine participants. The questionnaire started with 12 items and ended with 11 items, with the elimination of the question about the obligation for the preceptor to have a period of assistance exclusively before starting the trainer role, justified by the country having a low number of specialized professionals (Chart 4). The Figure shows the process for building the matrix and Charts 1 to 4 the number of cycles until the final matrix and scores (MRP-FCM Organization Matrix based on the judges’ evaluation) (3rd stage).

**Chart 4:** Criterion excluded from matrix.

Desirable matrix in the program	How to organize and evaluate the criterion	Note
Hiring professionals already with a minimum period of assistance prior to preceptorship work (“flight hours”).	Number of family and community medical professionals with at least 3 years of experience *before* becoming a preceptor.	Excluded in cycle 2, 44.4% of the judges agreed to maintain this criterion, thus, it was excluded for not reaching 80% agreement to maintain. Also suggested to take out in the comments due to the low number of professionals specializing in FCM in 2021.

FCM: family and community medicine.

**Figure:**
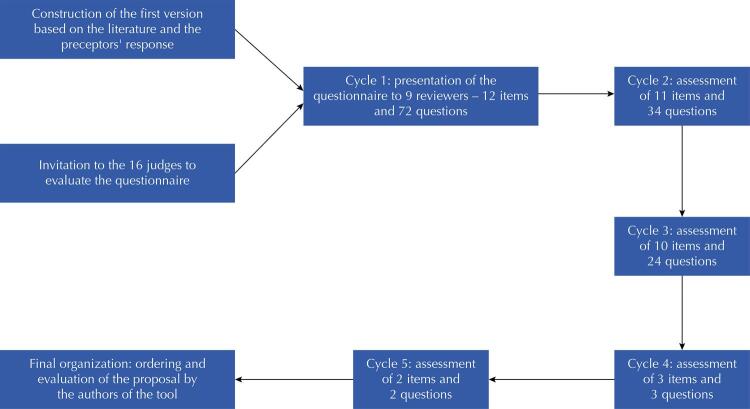
Conducting evaluation cycles of the Delphi questionnaire to define the matrix of residency programs in FCM.

FCM: family and community medicine.

The instrument was divided into Unit Organization (Chart 1), Human Resources (Chart 2), and Preceptor-Resident Relationship (Chart 3). Each item received a minimum value of 1 and a maximum of 10 according to the relevance given by the judges, with most items receiving a maximum score of 8 or 9. Only two items received a maximum score: the preceptor’s obligation to be a specialist in FCM and the payment of a supplementary scholarship to professionals who work with concomitant preceptorship. Charts 1 to 4 present the final instrument.

**Chart 1:** Group 1: organization of the unit (A.1–A.3).

Desirable matrix in the program	How to organize and evaluate the criterion	Number of evaluation cycles (1–5) and weighting defined by the judges (8–10)
A. 1. Adequacy of the unit’s physical space for work.		
A.1.1 Number of offices suitable for the entire team in service, without the need for rotations during the periods that the resident is in consultation. Considering that they also has activities outside the office.	At least one office per professional and/or no need for rotations.	4 cycles and reaching a weighting of 9 points.
A.1.2 Rooms and materials for procedures in the unit (activities proposed by the Competency-Based Curriculum – CBC).	Room to carry out training procedures for the specialty based on the CBC, whether in the reference unit or in another space that guarantees training (such as another unit).	
If these activities are not available in the unit, the resident has spaces on the grid to perform in other training spaces.		
A.2. Improving the organization of the agenda and demand for primary care services in the context of school-units, considering the social context and vulnerability of the defined population.	Residents have a protected agenda to attend and discuss cases. In addition, they have spaces for home visits, procedures, community groups.	3 cycles, reaching a weighting of 9 points.
(Note: both the preceptor and the resident must have protected spaces for adequate training of the professional in training/preceptor, considering the uniqueness of FCM (home visits, collective activities, unit meetings, theoretical agenda, among other specific demands of each program). The unique contexts of each program should also be considered: academic, in the SUS network and in the private network).		
A.3. Qualification and better interrelation between service management and medical residency so that the unit remains a school-unit.	Management cooperates with the organization of the unit so that adequate training is obtained, agreeing on goals and promoting feedback spaces for the unit’s professionals and users with medical residency.	3 cycles, reaching a weighting of 8 points.

FCM: family and community medicine; SUS: Sistema Único de Saúde.

**Chart 2:** Group 2: human resources (B.1–B.4).

Desirable matrix in the program	How to organize and evaluate the criterion	Number of cycles (1–5) and weighting defined by the judges (8–10)
B.1. Encourage and give preference to hiring professionals for the position of preceptorship with a degree and/or residency in family and community medicine with desire/training in preceptorship.	All professionals are FCM experts.	2 cycles, with 10 points of final value.
B.2. Continuous improvement of these professionals through.		
B.2.1. In-service education based on permanent education strategies and demands: the unit, the service portfolio, and the residency program.	Training process of the preceptor based on the demands of the service, residency and unit, having a protected space in the agenda for this.	3 cycles and 9 weighting points.
B2.2. Continuing Education of the preceptor through preceptorship courses, health education, continued clinical training, case matrix support, among others. Also the inclusion of permanent education in teams that have residents and preceptors, with a protected agenda.	Release of the preceptor for health department courses, external courses, continuing education through the program, matrix support with other professionals, among others.	3 cycles and 9 weighting points.
B.3. Provide ongoing training to professionals (preceptors, residents and residency unit staff) on the specialty, its updates and characteristics, so that everyone has the same discourse and can advance as the specialty advances.	Are residents, preceptors and staff systematically presented with information and training on the specialty?	5 cycles and 9 weighting points.
B.4. Financial support for preceptors, with supplementary salary or preceptorship scholarship.	Evaluate whether the preceptor has a differentiated salary for the position compared to exclusive assistant physicians (salary supplementation).	3 cycles and 10 weighting points.

**Chart 3:** Group 3: preceptor-resident relationship (C.1–C.2).

Desirable matrix in the program	How to organize and evaluate the criterion	Number of cycles (1–5) and weighting defined by the judges (8–10)
C.1. Careful observation of the number of residents per preceptor to have an adequate training moment in accordance with the organization of the program based on the conformation of the team.	Presence of a maximum of 3 residents per preceptor, adding 1st and 2nd year residents (40h).	4 cycles and 9 weighting points.
C.2. Observation of the overload of “learners” per preceptor at the undergraduate and residency levels.	The maximum number is 5 students (undergraduates and residents). *It may be suitable depending on the possibility of the team, space, agreements with educational institutions (not generating preceptor overload).	5 cycles and 9 weighting points.

FCM: family and community medicine.

## DISCUSSION

The results of the work show that most of the preceptors in the state of São Paulo are specialists in the area and trained for the position of educators. Their main difficulties in exercising the position are related to organizing the agenda, the unit and the relationship with municipal managers, who often have different objectives in relation to the function of medical residency, seeing the resident as a team doctor and not a doctor in training. Similar data were perceived in other works on residency in FCM^14,15^.

From the preceptors’ words and the current legislation, recommendations are proposed applicable to medical residency programs in Family and Community Medicine, which are validated by judges, resulting in a matrix with 11 items, organized into 3 cores: unit organization, humans resource, and preceptor-resident relationship. This makes it possible to observe training from a more realistic perspective compared to what is currently done, as carried out by multidisciplinary residency in health^16^. Thus, the instrument includes the structure and training processes, adapted from Donabedian’s triad and the preceptors’ experience^12^.

When observing the instrument, the first evaluated group (A1 to A3) are the unit’s physical and organizational resources, of which physical space, the possibility of developing the expected competencies in the residency, and the organization of the unit are evaluated, aspects that directly influence resident training. Unlike hospital residencies, in which the service structure is a rotating one, PHC is characterized by being solution-oriented, close to individuals (accessible), with a community focus and a multidisciplinary team^17^, with educational training in PHC also unique, as demonstrated in other studies^18,19^.

The above points were listed as challenges by the preceptors in the state of São Paulo in working on the residency and coincide with those found in previous research^1,15,20,21^, being corroborated by the judges as points to be observed in the program. This is a critical point that demonstrates the need for dialogue between teaching and assistance.

When evaluating domain A, the three components converge to two essential points: population size and physical structure of the residency unit/program, since they delimit the appointment schedule, health unit activities, and teaching-learning scenarios and methodologies, in addition to the health and residency financing itself. Thus, the adaptation of learning scenarios interferes with the organization of municipal health departments due to the needs of population coverage, health financing, adequacy of physical space and health teams, including the legislation that governs such aspects ^6,17,22^.

The adequate population size per team in “school-units” is suggested since 2015 as a maximum of 3,000 people, in addition to moments of permanent education for both the preceptor and the resident and what would be their functions in the unit^23^. However, the presence of these challenges demonstrates that it is necessary to improve the dialogue and definition of work processes between residency and municipal management, as other works have already demonstrated that the objectives are different^14,15^, making it difficult for the MRP-FCM to achieve its objectives: education based on quality service and training model.

Therefore, component A of the recommendations is mandatory, since it is necessary to observe the unit and how it is integrated into the municipality, from the population covered to the work process of resident training.

It is known that from the genesis of legislation to its effective implementation, several steps are necessary with constant monitoring to adapt the structure and process. Thus, the presented matrix can be a strategy to achieve this objective, as carried out in the urgency and emergency network^24^.

Another learning scenario that can be inserted in training is the simulation labs. By its nature, FCM requires simulations involving the behavioral domain (such as those developed with actors) and technical skills (such as placement of an intrauterine device), being a new learning strategy to be acquired by preceptors and a new scenario for residency^25,26^.

In the second group of questions (B1 to B4), involved in the Human Resources domain, two items had a high acceptance value by the judges: if the preceptor is a specialist in FCM (B1) and if the preceptor receives a differentiated value in their salary (B4).

In 2020, there were 7,149 specialist professionals in the field, with an increase of 3,127 professionals in the last 5 years^3,27^. Despite the judges’ desire for specialists in the field to train residents, the national policy for medical residency in FCM allows the activity of professionals from other areas as preceptors^23^, possibly because of the low number of specialists. With this, it is mandatory to assess whether the residency preceptors are specialists in the field or if they have the competence to exercise the position, which is contemplated by the instrument developed.

The second item that received maximum weighting was scholarship supplementation to be a preceptor (B4). This is an item that the Ministry of Health has encouraged since 2019, with remuneration for the municipality and the preceptor^22^. However, although studies show that preceptors do not routinely receive scholarships^15,28^, there is already evidence that this is not the main motivation for professionals to exercise this function^28^. Furthermore, the use of any financial incentive must be coupled with production indicators for it to be an effective inducing policy. However, the judges did not specify the reason for their choice, which prevents an assertive conclusion on the topic and could be an opportunity to improve the instrument in future versions.

The other items in group B address the aspect of education through work, permanent education and continuing education, being professional qualification health policies^29^. When observing the preceptor, many do not clearly and homogeneously define the FCM specialty^28^. Thus, it is mandatory that the program systematically present the specialty to all those involved, ensuring adequate knowledge of the field (items B2 and B3).

Furthermore, considering Permanent Education as a health policy, its presence in the residency is essential^30^. Finally, training as an educator provides preceptors with a greater quantity and quality of educational and care tools^25^(B2).

The third evaluation domain (C1 and C2) required the most evaluation cycles by the judges, as it aimed to reach a consensus on the number of students per preceptor, a value already determined by the National Commission for Medical Residency, with 6 residents per preceptor 40 hours^31^.

Despite this predetermined number, the programs vary greatly, with proportions ranging from 1 to 9 residents per preceptor in the state of São Paulo. In addition to residents, 67% of preceptors are responsible for undergraduates and other non-medical areas, increasing the number of trainees per professional^28^.

Such data correspond to significantly higher numbers as compared to programs abroad^28^. Although a lower resident-to-preceptor ratio is likely to be adequate, until the desired number of preceptors is reached, this item will continue to differ across programs. The repeated use of the instrument may provide data on the evolution of this indicator (preceptor/resident ratio) and its comparison between the regions, allowing intervention with the responsible instances for the continuous improvement of the programs.

Also in this domain, the number of students per preceptor is presented, resulting from the sum of FCM residents and students from other instances (undergraduate and multidisciplinary residency). It is important to observe this number because the more students who are under the tutelage of the preceptor, the less time dedicated to the residency and to the student.

Finally, the item excluded from the initial questionnaire, following the judges’ advice, was “Hiring professionals already with a minimum period of assistance prior to preceptorship work (“flight hours”)”, with at least 3 years of experience before preceptorship as the source of verification, as described for residency in Portugal^32^.

The justification is that the country still does not have a sufficient number of specialist professionals in the field to adequately provide assistance, teaching, management and preceptorship. Thus, it is necessary for the recent residency graduate to already take on a team to train new residents, which actually happens in practice^28^.

As limitations of this study, we can point out that the instrument was developed using as a basis only preceptors from the state of São Paulo, which is the state with the highest number of FCM programs implemented in stage 1. Stages 2 and 3 were nationwide in scope. It will still be necessary to validate the instrument in other federation states with different realities. It is also important to emphasize that any evaluation process should not be based on just one tool, but a set of tools^33^.

## CONCLUSION

This work proposes an evaluative instrument of structures and processes for FCM residency programs divided into three domains: Unit Organization, Human Resources and Preceptor-Resident Relationship. Such an instrument, when properly validated, may allow the continuous evaluation of programs to ensure the implementation of public policies that govern FCM residency.
