# Supplementary material for: Improving the Quality of Residency Programs in Family and Community Medicine
Source: Rev Saude Publica. 2023 Sep 14;57:65. doi: 10.11606/s1518-8787.2023057004822 (PMC10519678; doi:10.11606/s1518-8787.2023057004822)
Supplement: Translation

# Translation: Aprimorando a qualidade de Programas de Residência em Medicina de Família e Comunidade

**Authors:** Lucas Gaspar Ribeiro, Eliana Golbfarb Cyrino, Antônio Pazin-Filho

**Affiliations:** I Brasil Universidade de São Paulo. Faculdade de Medicina de Ribeirão Preto. Departamento de Clínica Médica. Ribeirão Preto, SP, Brasil; II Brasil Universidade Estadual Paulista “Júlio de Mesquita Filho”. Faculdade de Medicina de Botucatu. Departamento de Saúde Pública. Botucatu, SP, Brasil

**Keywords:** Medicina de Família e Comunidade, Internato e Residência, Avaliação de Programas e Instrumentos de Pesquisa

## Abstract

**OBJETIVO:**

Desenvolver e apresentar um instrumento para avaliar e monitorar a qualidade de programas de residência médica em medicina de família e comunidade (MFC) a partir dos preceptores e residentes, considerando a inserção do programa de rede de saúde.

**MÉTODO:**

A elaboração do instrumento foi desenvolvida em três etapas: 1) entrevista com preceptores em MFC; 2) revisão da literatura; e 3) produção, adequação e aprovação do instrumento avaliativo por profissionais renomados na MFC brasileira. A terceira etapa contou com nove pessoas e utilizou a técnica Delphi com obtenção de 80% de concordância. Para os resultados qualitativos foi utilizada a análise de conteúdo de Bardin.

**RESULTADOS:**

Ao todo ocorreram cinco ciclos de avaliação para adequação das recomendações propostas, com eliminação de um item e ponderação, e metodologia de análise de resultado de dez itens resultantes. Atingiu-se uma matriz esperada para a organização de programas de residência na rede de saúde, dividida em três domínios: organização da unidade, recursos humanos e relação preceptor-residente.

**CONCLUSÃO:**

Um instrumento de avaliação e monitoramento de programas de residência em MFC pode ser uma ferramenta para auxiliar gestores de programas e permite a avaliação e monitoramento, qualificando-os continuamente.

## INTRODUÇÃO

A residência médica é considerada a melhor estratégia para formação de novos especialistas médicos, considerada uma formação após a graduação. Alguns autores relatam que teve início nos Estados Unidos com as clínicas médica e cirúrgica, no final do século XIX. No Brasil, ela é considerada legalmente como curso de pós-graduação, modalidade de especialização, com foco no treinamento em serviço, existindo desde 1944^1^.

As residências em medicina de família e comunidade (MFC) iniciaram em 1976^1^ e apresentaram uma expansão pequena de vagas até 2013 em comparação com o período seguinte (2013–2021)^2^. A partir da Lei 12.871/2013, conhecida como Lei Mais Médicos, o número de vagas ocupadas passou de 206 em 2011 para mais de 2.282 (aumento de 11,7 vezes) – dados de outubro de 2021. O estado de São Paulo possuía 62 programas de residência médica (PRM) em MFC cadastrados, com 37 programas com residentes (405 residentes e 1.366 vagas, 29% de ocupação), correspondendo a 17,8% dos residentes em MFC do Brasil, sendo o estado com maior número^3^ no ano de 2021. Os dados do ano de 2021 foram obtidos a partir da Lei de Acesso à Informação^4^.

Por mais que a Estratégia Saúde da Família (ESF) esteja pautada na presença do especialista em MFC como profissional médico, há aproximadamente 7 mil profissionais de MFC^3^ para mais de 43 mil equipes de Saúde da Família no país^5^. Essa discrepância se intensifica quando observada a distribuição desses profissionais, presentes em sua maioria no Sul e Sudeste, correspondendo a 71,4% dos especialistas do Brasil e 46% das unidades de saúde da família^3,5^.

Amapá, Bahia, Maranhão e Piauí apresentam menos de 1,1 especialista em MFC por 100 mil habitantes, enquanto Acre, Distrito Federal, Rio Grande do Sul e Santa Catarina têm entre 4,9 e 8,7 especialistas por 100 mil habitantes^3^. Para fazer o cálculo da relação ideal, é possível inferir que cada médico de família e comunidade é responsável por 4 mil pessoas^6^, ou seja, o país necessitaria da relação de 25 médicos de família e comunidade por 100 mil habitantes. Assim, considerando que no ano de 2020 foram formados aproximadamente 1.500 novos especialistas pela residência, mantendo o atual volume de vagas ocupadas, o país só atingiria o número de especialistas necessários após duas décadas, sem considerar perdas ao longo do tempo.

Perante a escassez e concentração de especialistas, a formação depende de preceptores de outras áreas de atuação, os quais são legalmente habilitados para tal papel, segundo a legislação específica dos PRM-MFC, diferentemente de outras áreas, que exigem especialidade na área de formação como pré-requisito para a função^7^.

Isso provavelmente implica grande variabilidade nas condições de formação. Soma-se a essa situação a existência de informações e padrões para condições mínimas de uma estrutura física e insumos adequados para garantir a formação. Com isso, os programas necessitam de diretrizes para a abertura e expansão de vagas de forma ordenada e capaz de suprir a formação, considerando a singularidade da MFC, que atua predominantemente na ESF/atenção primária à saúde (APS).

Para tal, é necessário compreender o próprio serviço e avaliá-lo. Esse ato consiste em emitir um juízo de valor a partir de um padrão predeterminado ou um referencial ideal, buscando falhas e corrigindo-as^8^. Donabedian^11^ reforça que o processo de avaliar exige um monitoramento contínuo do serviço de saúde, buscando detectar e corrigir desvios de padrões, e pode avaliar estruturas, processos e resultados obtidos. Diante desses fatores, este estudo propôs uma matriz para avaliação e monitoramento dos PRM-MFC no Brasil, com enfoque na APS.

## MÉTODOS

Trata-se de um estudo de metodologia avaliativa, descritiva e exploratório iniciado em 2018 e constituído de pesquisa quantitativa e qualitativa, com uso de formulário eletrônico para obtenção de respostas dos participantes e revisão de literatura para complementação e caracterização do instrumento apresentado ao final.

Este estudo desenvolveu um instrumento matriz para definir as condições de formação ofertadas pelos PRM-MFC com base na estrutura da APS. A construção foi dividida em três estágios de desenvolvimento, buscando inicialmente compreender a realidade na perspectiva do preceptor e finalizando com a proposição de um instrumento avaliativo com possibilidade de aplicação em âmbito nacional.

No primeiro estágio, foram convidados 132 preceptores em MFC do estado de São Paulo, entre fevereiro e novembro de 2018, aplicando-se um formulário do Google com questões abertas e fechadas. Todos os profissionais foram convidados via correio eletrônico, com o objetivo de compreender características do participante e do trabalho que realiza. Os resultados completos estão em artigo específico^12^, no qual foram enumeradas as forças, oportunidades, fraquezas e ameaças (FOFA) que 67 preceptores percebem em seu trabalho e nos PRM-MFC.

Com as respostas obtidas, o segundo estágio iniciou com a revisão de literatura nacional e internacional, resoluções e portarias sobre residência médica em geral e de MFC, comparando a literatura com a percepção dos preceptores e sobrepondo a FOFA com a tríade de Donabedian^12^. O quadro construído permitiu obter as respostas passíveis de avaliação na APS e gerar uma matriz de recomendações sobre PRM-MFC, finalizando o segundo estágio da matriz a ser validada nacionalmente.

O terceiro estágio validou por pares o documento, garantindo a homogeneidade de qualidade, com possibilidade de uso em todas as regiões. Para tanto o instrumento foi apresentado a um grupo de juízes especialistas em MFC, utilizando-se o método Delphi^13^. Foram convidados a participar os presidentes de todas as associações estaduais ativas de MFC e o presidente da Sociedade Brasileira de Medicina de Família e Comunidade, contemplando 24 pessoas. Eles foram escolhidos pelos autores considerando sua diversidade regional e representatividade nacional. O presidente da estadual paulista foi excluído por ser o autor do projeto.

O questionário Delphi possuía estrutura igual para todas as questões: descrição do item avaliado, perguntas sobre a pertinência e permanência do item no questionário final (sim e não), escalas Likert de 0 a 10 sobre a relevância do item e que nota ou valor esse item deveria receber na avaliação do programa (0 a 10). Também foram inseridas perguntas abertas sobre sugestões de escrita, comentários sobre a nota e sobre o tema em questão em todos os itens para aperfeiçoá-los. Para manutenção dos itens, deveria haver consenso entre os juízes, sendo considerado o valor de corte de 80%, valores menores acarretariam a não aprovação do item.

Para as questões abertas, com sugestão do texto e fontes de verificação, foi utilizada a análise de Bardin, permitindo adequar a escrita, justificar eliminações e fundir itens, se necessário.

Ambas as pesquisas, tanto com preceptores, quanto com os juízes, foram aprovadas por comitês de ética em pesquisa, sob os protocolos CAAE 78853317.0.0000.5411 e 30805420.5.0000.5440.

Ao final dos ciclos de discussão, as recomendações foram reavaliadas pelos autores, organizadas e cotejadas com a legislação vigente.

## RESULTADOS

As respostas da fase 1 demonstraram que 70% dos entrevistados eram especialistas em MFC e mais de 90% tinham alguma formação específica em preceptoria. Embora 62% (42 indivíduos) tenham se considerado bastante satisfeitos com sua atuação, 27 pessoas relataram grande dificuldade no trabalho.

As recompensas apresentadas pelos preceptores com maior frequência são: qualificação na área, atualização, qualificação pessoal e participação em um processo de transformação. Os desafios foram: demanda excessiva *versus* dificuldade no tempo de ensino, subvalorização da especialidade, dificuldade na relação com a gestão local e dificuldade no processo de ensino, espaço físico insuficiente para educação e dificuldade na organização da agenda da unidade.

Associando a visão do preceptor com a literatura nacional e internacional sobre residência médica e na área em específico, foi produzida a matriz de recomendações a PRM-MFC (estágio 2). Ela foi apresentada aos juízes para a continuidade do processo de construção. Ao todo, foram necessários cinco ciclos de avaliação entre os juízes até obter a concordância de pelo menos 80%, com nove participantes. O questionário tinha inicialmente 12 itens e foi finalizado com 11, com a eliminação da questão sobre a obrigatoriedade de o preceptor ter um período de assistência exclusiva antes de iniciar o papel de formador, o que se justificou pelo país ter baixo número de profissionais especialistas (Quadro 4). O processo para a construção da matriz está apresentado na Figura e o número de ciclos até a matriz final e pontuações nos quadros 1 a 4 (Matriz de organização do PRM-MFC baseada na avaliação dos juízes) (3º estágio).

**Quadro 4:** Critério excluído da matriz.

Matriz desejável no programa	Modo de organizar e avaliar o critério	Observação
Contratação de profissionais com um período mínimo de assistência prévia ao trabalho de preceptoria (“horas de voo”).	Número de profissionais médicos de família e comunidade com pelo menos três anos de atuação *antes* de serem preceptores.	Apesar de 44,4% dos juízes concordarem em manter esse critério, ele foi excluído no ciclo 2 por não atingir 80% de concordância em sua manutenção. Também foi sugerido retirar dos comentários por baixo número de profissionais especialistas em MFC no ano de 2021.

MFC: medicina de família e comunidade.

**Figura:** Realização dos ciclos de avaliação do questionário Delphi para definir a matriz dos programas de residência em MFC.

MFC: medicina de família e comunidade.

O instrumento foi dividido em organização da unidade (Quadro 1), recursos humanos (Quadro 2) e relação preceptor-residente (Quadro 3). Cada item recebeu um valor mínimo de 1 e máximo de 10, de acordo com a relevância dada pelos juízes, e a maioria dos itens recebeu nota máxima 8 ou 9. Apenas dois itens receberam nota máxima: a obrigatoriedade de o preceptor ser especialista em MFC e o pagamento de bolsa complementar a profissionais que atuam com preceptoria concomitante. Os quadros 1 a 4 apresentam o instrumento final.

**Quadro 1:** Grupo 1: organização da unidade (A.1–A.3).

Matriz desejável no programa	Modo de organizar e avaliar o critério	Número de ciclos avaliatórios (1–5) e ponderação definida pelos juízes (8–10)
A.1. Adequação de espaço físico da unidade para o trabalho		
A.1.1 Número de consultórios adequados para toda a equipe em atendimento, sem necessidade de rodízios durante os períodos em que o residente está em consulta, considerando que ele também tem atividades fora do consultório.	Ao menos um consultório por profissional e/ou não necessidade de rodízios.	4 ciclos e atingindo a ponderação de 9 pontos.
A.1.2 Salas e materiais de procedimentos na unidade (atividades propostas pelo currículo baseado em competências – CBC).	Sala para realizar os procedimentos formativos para a especialidade baseado no CBC, seja na unidade de referência, seja em outro espaço que garante sua formação (como, por exemplo, em outra unidade).	
Caso não tenha essas atividades na unidade, o residente deve ter espaços na grade para realizar em outros espaços de treinamento.		
A.2. Aprimoramento da organização da agenda e demanda dos serviços de atenção primária no contexto de unidades-escola, considerando o contexto social e vulnerabilidade da população definida.	O residente tem agenda protegida para atender e discutir casos. Além disso, tem espaços para visita domiciliar, procedimentos e grupos comunitários.	3 ciclos, atingindo a ponderação de 9 pontos.
(Obs.: tanto o preceptor quanto o residente devem ter espaços protegidos para a formação adequada do profissional em treinamento/preceptor, considerando a singularidade da MFC (visitas domiciliares, atividades coletivas, reuniões da unidade, agenda teórica, dentre outras demandas específicas de cada programa). Também devem ser considerados contextos singulares de cada programa: acadêmico, em rede do Sistema Único de Saúde (SUS) e em rede privada.		
A.3. Qualificação e melhor inter-relação da gestão do serviço com a residência médica para que se mantenha a unidade como unidade-escola.	A gestão corrobora a organização da unidade para que seja obtida formação adequada, pactuando metas e promovendo espaços de feedback dos profissionais da unidade e usuários com a residência médica.	3 ciclos, atingindo a ponderação de 8 pontos.

MFC: medicina de família e comunidade.

**Quadro 2:** Grupo 2: recursos humanos (B.1–B.4).

Matriz desejável no programa	Modo de organizar e avaliar o critério	Número de ciclos (1–5) e ponderação definida pelos juízes (8–10)
B.1. Incentivar e dar preferência para a contratação de profissionais para o cargo de preceptoria com título e/ou residência em medicina de família e comunidade com desejo/formação em preceptoria.	Todos os profissionais são especialistas em MFC.	2 ciclos, com 10 pontos de valor final.
B.2. Aprimoramento contínuo desses profissionais		
B.2.1. Educação em serviço baseado em estratégias de educação permanente e de demandas: da unidade, da carteira de serviços e do programa de residência.	Processo formativo do preceptor baseado nas demandas do serviço, da residência e da unidade, com um espaço protegido na agenda para tal.	3 ciclos e 9 pontos de ponderação.
B2.2. Educação continuada do preceptor por meio de cursos de preceptoria, educação em saúde, formação clínica continuada, matriciamento de casos, dentre outras. Também a inclusão de educação permanente em equipes que tenha residentes e preceptores, com agenda protegida.	Liberação do preceptor para cursos da secretaria de saúde, cursos externos, formação continuada pelo programa, matriciamento com outros profissionais, dentre outros.	3 ciclos e 9 pontos de ponderação.
B.3. Oferecimento de formação continuada aos profissionais (preceptores, residentes e equipe da unidade da residência) sobre a especialidade, suas atualizações e características, para que todos tenham o mesmo discurso e consigam avançar conforme o avanço da especialidade.	Há sistematicamente a apresentação aos residentes, preceptores e equipe informação e formação sobre a especialidade?	5 ciclos e ponderação de 9 pontos.
B.4. Apoio financeiro para os preceptores, com complementação de salário ou bolsa de preceptoria.	Avaliar se o preceptor tem um salário diferenciado para o cargo em comparação a médicos assistencialistas exclusivos (complementação salarial).	3 ciclos e 10 de ponderação.

**Quadro 3:** Grupo 3 – Relação preceptor-residente (C.1–C.2)

Matriz desejável no programa	Modo de organizar e avaliar o critério	Número de ciclos (1–5) e ponderação definida pelos juízes (8–10)
C.1. Observação criteriosa do número de residentes por preceptor para ter adequado momento de formação de acordo com a organização do programa baseado na conformação da equipe.	Presença de, no máximo, três residentes por preceptor, somando os residentes do 1º e 2º ano (40h).	4 ciclos e 9 de ponderação.
C.2. Observação da sobrecarga de “educandos” por preceptor nos níveis de graduação e residência.	O número máximo é cinco educandos (graduandos e residentes). * Pode ser adequado a depender da possibilidade da equipe, espaço, acordos com as instituições de ensino (não gerando sobrecarga ao preceptor).	5 ciclos e 9 de ponderação.

MFC: medicina de família e comunidade.

## DISCUSSÃO

Os resultados do trabalho demonstram que em sua maioria os preceptores do estado de São Paulo são especialistas na área e têm formação para o cargo de educadores. Suas principais dificuldades em exercer o cargo estão relacionadas à organização da agenda e da unidade, bem como à relação com os gestores municipais. Estes muitas vezes têm objetivos diferentes em relação à função da residência médica, observando o residente como um médico da equipe e não um médico em formação. Dados semelhantes foram percebidos em outros trabalhos sobre residência em MFC^14,15^.

Considerando a fala dos preceptores e a legislação atual, propõem-se recomendações aplicáveis aos PRM-MFC que foram validadas por juízes, resultando em uma matriz com 11 itens, organizados em três núcleos: organização da unidade, recursos humanos e relação preceptor-residente. Isso permite observar a formação com uma óptica mais realística em comparação ao que é realizado atualmente, como o que ocorre na residência multiprofissional em saúde^16^. Assim, o instrumento contempla a estrutura e processos de formação, adaptados da tríade de Donabedian, e a vivência dos preceptores^12^.

Quando observado o instrumento, o primeiro grupo avaliado (A1 a A3) contempla os recursos físicos e organizativos da unidade, dos quais são avaliados o espaço físico, a possibilidade de desenvolver as competências esperadas na residência e a organização da unidade, aspectos que influenciam diretamente a formação do residente. Diferentemente de residências hospitalares, em que a estrutura do serviço é de rotatividade, a APS tem como características ser resolutiva, próxima dos indivíduos (acessível), com enfoque comunitário e em equipe multirpofissional^17^, sendo a formação educacional na APS também singular, como demonstrado em outros trabalhos^18,19^.

Esses pontos foram elencados pelos preceptores do estado de São Paulo como desafios de atuar na residência e coincidem com os achados de pesquisas anteriores^1,15,20,21^, sendo corroborados pelos juízes como pontos a serem observados no programa. Esse é um ponto crítico que demonstra a necessidade da interlocução ensino-assistência.

Quando avaliado o domínio A, os três componentes convergem para dois pontos essenciais: tamanho populacional e estrutura física da unidade/programa de residência, pois delimitam a agenda de consultas, atividades da unidade de saúde e cenários e metodologias de ensino-aprendizagem, além do próprio financiamento da saúde e da residência. Assim, a adequação de cenários de aprendizagem se interpõe com a própria organização das secretarias municipais de saúde pelas necessidades de cobertura populacional, financiamento em saúde, adequação de espaço físico e equipes de saúde, inclusive pela legislação que rege tais aspectos^6,17,22^.

O tamanho populacional adequado por equipe em “unidades-escola” sugerido desde 2015 é o máximo de 3 mil pessoas. Além disso, recomendam-se momentos de educação permanente para o preceptor e para o residente e a delimitação de suas funções na unidade^23^. Contudo, a presença desses desafios demonstra que é preciso aprimorar a interlocução e definição de processos de trabalho entre a residência e a gestão municipal, pois já foi demonstrado por outros trabalhos que os objetivos são diferentes^14,15^, dificultando que o PRM-MFC atinja seu objetivo: educação baseada no serviço de qualidade e modelo formativo.

Sendo assim, o componente A das recomendações é mandatório, pois é preciso observar a unidade e como ela está inserida no município, desde a população coberta até o processo de trabalho da formação do residente.

É conhecido que desde a gênese de uma legislação até sua efetiva implementação várias etapas são necessárias com monitorização constante para adequar a estrutura e processo. Assim, a matriz apresentada pode ser uma estratégia para atingir esse objetivo, como realizado na rede de urgências e emergências^24^.

Outro cenário de aprendizagem que pode ser inserido na formação são os laboratórios de simulação. Por sua natureza, a MFC necessita de simulações que envolvam o domínio comportamental (como aquelas desenvolvidas com atores) e habilidades técnicas (como colocação de dispositivo intrauterino), sendo uma nova estratégia de aprendizagem a ser adquirida pelos preceptores e novo cenário para a residência^25,26^.

No segundo grupo de questões (B1 a B4), envolvido no domínio recursos humanos, dois itens apresentaram alto valor de aceitação pelos juízes: o preceptor é especialista em MFC (B1) e o preceptor recebe um valor diferenciado em seu salário (B4).

No ano de 2020 existiam 7.149 profissionais especialistas na área, ocorrendo um aumento de 3.127 profissionais nos últimos 5 anos^3,27^. Apesar de os juízes apontarem o desejo de que haja especialistas na área para a formação de residentes, a política nacional de residência médica em MFC permite a atividade de profissionais de outras áreas como preceptores^23^, uma justificativa pode ser o baixo número de especialistas. Com isso, é mandatório avaliar se os preceptores da residência são especialistas na área ou têm competência para exercer o cargo, o que está contemplado pelo instrumento desenvolvido.

O segundo item que recebeu ponderação máxima foi a complementação de bolsa para ser preceptor (B4), algo que o Ministério da Saúde incentiva desde 2019, com a remuneração para o município e para o preceptor^22^. No entanto, apesar de estudos demonstrarem que o preceptor não recebe bolsa rotineiramente^15,28^, já há evidências de que essa não é a principal motivação para que os profissionais exerçam essa função^28^. Ademais, a utilização de qualquer incentivo financeiro deve estar relacionada a indicadores de produção para que seja uma política indutora efetiva. Entretanto, os juízes não especificaram o motivo de sua escolha, o que impede uma conclusão assertiva sobre o tema e poderia ser uma oportunidade de aprimoramento do instrumento em versões futuras.

Os outros itens do grupo B visam o aspecto de educação pelo trabalho, educação permanente e educação continuada, sendo políticas de saúde de qualificação profissional^29^. Quando aos preceptores, muitos não definem de forma clara e homogênea a especialidade MFC^28^. Assim, é mandatório que o programa apresente sistematicamente a especialidade a todos os envolvidos, garantindo adequado conhecimento da área (itens B2 e B3).

Além disso, considerando a educação permanente como política de saúde, sua presença na residência é fundamental^30^. Por fim, a formação como educador possibilita ao preceptor maior quantidade e qualidade de ferramentas educacionais e assistenciais^25^ (B2).

O terceiro domínio (C1 e C2) foi o que mais exigiu ciclos de avaliação pelos juízes, pois visou atingir um consenso do número de educandos por preceptor, valor já determinado pela Comissão Nacional de Residência Médica, sendo seis residentes por preceptor com carga de 40 horas^31^.

Apesar desse número predeterminado, os programas apresentam uma grande variação, com proporções que abrangem entre um e nove residentes por preceptor no estado de São Paulo. Além dos residentes, 67% dos preceptores são responsáveis por graduandos e outras áreas não médicas, aumentando o número de formandos por profissional^28^.

Tais dados correspondem a números expressivamente superiores quando comparados a programas do exterior^28^. Apesar de uma proporção menor entre residentes por preceptor provavelmente ser adequada, até que se atinja um número desejado de preceptores, esse item continuará a ser divergente entre os programas. A utilização repetida do instrumento poderá prover dados sobre a evolução desse indicador (relação preceptor-residente) e sua comparação entre as regionais, permitindo intervenção nas instâncias responsáveis para aprimoramento contínuo dos programas.

Também nesse domínio é apresentado o número de educandos por preceptor, resultado da soma de residentes de MFC e alunos de outras instâncias (graduação e residência multidisciplinar). É importante observar esse número porque quanto mais educandos estiverem sob a tutela do preceptor, menor pode ser o tempo dedicado à residência e ao graduando.

Por fim, o item excluído do questionário inicial por orientação dos juízes foi “Contratação de profissionais já com um período mínimo de assistência prévia ao trabalho de preceptoria (“horas de voo”)”, sendo a fonte de verificação de pelo menos três anos de atuação antes da preceptoria, como descrito para a residência em Portugal^32^.

A justificativa é que o país ainda não possui um número suficiente de profissionais especialistas na área para ter assistência, docência, gestão e preceptoria de forma adequada. Assim, é necessário que o recém-formado da residência já assuma uma equipe para formar novos residentes, o que realmente ocorre na prática^28^.

Como limitações do estudo podemos apontar que o instrumento foi desenvolvido utilizando como base apenas os preceptores de São Paulo, que é o estado com maior número de programas de MFC implantados na etapa 1. Já as etapas 2 e 3 tiveram abrangência nacional. Ainda será necessário validar o instrumento em outras unidades da federação com realidades distintas. Também é importante ressaltar que qualquer processo de avaliação não deve usar apenas uma ferramenta, mas um conjunto^33^.

## CONCLUSÃO

Este trabalho propõe um instrumento avaliativo de estruturas e processos para PRM-MFC dividido em três domínios: organização da unidade, recursos humanos e relação preceptor-residente. Tal instrumento, quando adequadamente validado, poderá permitir a avaliação contínua dos programas para garantir a implementação das políticas públicas que regimentam a residência de MFC.
